# Innovation in surgery

**DOI:** 10.1093/bjs/znad164

**Published:** 2023-06-10

**Authors:** Oleksandr Khoma, Jerome M Laurence, Charbel Sandroussi, Bas P L Wijnhoven

**Affiliations:** Royal Prince Alfred Hospital Institute of Academic Surgery, University of Sydney, Sydney, New South Wales, Australia; Department of Medicine, University of Notre Dame, Sydney, New South Wales, Australia; Royal Prince Alfred Hospital Institute of Academic Surgery, University of Sydney, Sydney, New South Wales, Australia; Department of Upper Gastrointestinal Surgery, Royal Prince Alfred Hospital, Sydney, New South Wales, Australia; Royal Prince Alfred Hospital Institute of Academic Surgery, University of Sydney, Sydney, New South Wales, Australia; Department of Upper Gastrointestinal Surgery, Royal Prince Alfred Hospital, Sydney, New South Wales, Australia; Department of Upper Gastrointestinal Surgery, Erasmus MC, Rotterdam, Netherlands

## Abstract

The process of innovation is always valuable and can lead to great advances, both intended and unanticipated. However, the outcomes must always be carefully examined in terms of metric which considers more than could ever be tested in a RCT.

For the purpose of this discussion, a surgical innovation is taken to mean a new system, procedure, device or technology^1,2^. Here, the word ‘innovation’ refers to both a process and an outcome. The process of innovation is essential to progress, but it cannot be assumed that the outcome of any innovation is desirable *per se*^3^. Innovations are expected to be proven to be beneficial before widespread dissemination into the community^4^. However, universally agreed definitions of successful innovation are lacking. In clinical practice, success is quantified by some predefined metric (the main outcome measure). However, not everything that is important is measurable, and *vice versa*. For example, a medical administrator may measure the costs of the device used in a procedure, whereas the clinician could consider the postoperative visual analogue pain scores vastly more important. Not only are these outcomes different, they are not measured in a common unit. Studying the outcome of innovation in multiple non-commensurable metrics does not make that the societal value of these metrics is incomparable.

Desire to innovate implies a belief in the virtue of the process. However, it is impossible to predict the consequences of any innovation in practice. In biliary surgery, for example, because of a lower rate of incision-related morbidity with laparoscopic cholecystectomy (LC)^5^, and the increased availability of ultrasound imaging, the rate of cholecystectomy increased dramatically in the early 1990s^6^. Concurrently, the rate of serious complications (particularly bile duct injury) increased dramatically with the introduction of laparoscopy^7^. The implication of this simultaneous rising procedure rate and rising complication rate is seldom evaluated in terms of the absolute number of serious adverse events. Because bile duct injury and functional recovery are not commensurable, it can never be known, taking a utilitarian view of virtue^8^, whether the greatest happiness of humanity was served by the introduction of LC.

From a conventional scientific perspective, studies must analyse some outcome about which they can reasonably draw conclusions—the primary outcome by which the study is powered. In the case of LC, duration of hospital stay became the popular outcome measure. Less common, but arguably more important, outcomes may be very difficult to analyse. Perhaps if the key metric for LC was defined as rate of bile duct injuries, the procedure may have been consigned to the history books by 1990.

Another potential trap of current scientific methods is in allocating significance to the magnitude of change for any outcome of a novel intervention to be declared better, worse, or equal. Non-inferiority presumes equality (intervention OLD = intervention NEW). However, if the nominal outcome of intervention OLD is 10.01 and that of intervention NEW is 10.02, and the difference is below significance level, they are considered equal. In the next step of the innovation process, intervention NEWER is compared with intervention NEW, and is again found to be equal, although an advance of 0.01 again exists. Then study findings are interpreted as OLD = NEW = NEWER, although 10.03 is now significantly greater than 10.01, and an opportunity to detect benefit has been missed because OLD was never compared directly with NEWER. This ‘sensitivity phenomenon’ described by Poincaré^9^ becomes hazardous if sensitivity fails to detect inferiority below significance and 10.01 = 10 = 9.9, except in this example innovation was accepted as non-inferior because it was never compared with the original intervention. When intervention NEW becomes best practice, there is an ethical obligation to compare NEW and not OLD when examining intervention NEWER. For example, it would be considered unethical to perform an RCT of robotic *versus* open cholecystectomy today.

To better understand these ideas, a novel outcome measure for surgical innovations is proposed—net global utility. This is a composite outcome, which considers all potential effects of the innovation. This may include clinical, social, economic, and other consequences of the innovation. Net global utility also takes into account risk (probability × consequence). This metric attempts to resolve the issue of non-commensurability by not using any particular outcome measure in isolation. It is a subjective measure, which gives the observer licence to question the outcomes already chosen. Net global utility is comparable to the best available alternative, and derives its internal face validity from this property. Although the outcome is clearly subjective, no satisfactory conclusions about the real utility of any innovation can ever be drawn, even from the most rigorously conducted double-blind RCT, as it may not be until many years after the innovation has been adopted that a full understanding of its consequences can be known. The risks of highly improbable but very consequential adverse effects can be factored (the thalidomide effect)^10^ into the net global utility. The probability distribution of such events is not Gaussian, but fractal^11^. This means that great consequences can be anticipated, but with a probability that is very hard to define, which is almost certainly greater than would be derived from the Gaussian model. Additionally, the net global utility could encompass the incidental future benefits effect of innovation. An example is the transference of laparoscopic skills developed in LC to procedures that likely offer more benefit when performed laparoscopically (such as obesity surgery). One of the main advantages of net global utility is that it only requires knowledge of available metrics and principles of logical argument, providing an easy assessment tool to boost local oversight of introduction of innovation and policy-making, which is urgently needed^12^. Finally, net global utility could include an assessment of the innovation against a test of justice. For example, even if robotic cholecystectomy were to produce a better outcome for an individual patient, the impact of this on the access to care of other patients may be considered.

Net global utility includes some of the precautionary principle^13^. Some innovations carry a much lower risk of inferior outcomes where no alternative exists^14^. For example, the surgical checklist was a new practice (innovation) for which there was no competing alternative^15^. It carried low probability of causing harm with minimal financial costs—properties not shared by many other innovations in surgery. Most complex innovations are likely to have low net global utility; however, this may not be true in circumstances where it addresses a void in current practice. Introduction of electrosurgery can serve as an example of complex innovation with high net global utility^16^.

To exemplify the ideas more precisely, a graphical representation of three innovations is presented (*Fig. 1*). Innovation A is a highly complex alternative to an existing, much simpler system. The innovation may have one advantage (such as earlier hospital discharge), yet unappreciated risks (much higher chance of major bile duct injury). Initially, harm may be caused by the innovation (negative net global utility). Eventually surgical teams understand how to mitigate the risks and familiarity with the alternatives (open cholecystectomy) may wane, associated with the Dunning–Kruger effect (unjustified overconfidence in open cholecystectomy for example)^17^. As a result, the net global utility becomes positive. The positive net global utility may not be achieved necessarily by improvement in performance of the innovation, but by deterioration in performance of the alternative, a paradox of relative utility. Eventually another innovation may emerge from its own primary phase of negative net global utility, and so the net global utility of innovation A becomes negative again in comparison.

**Fig. 1:**
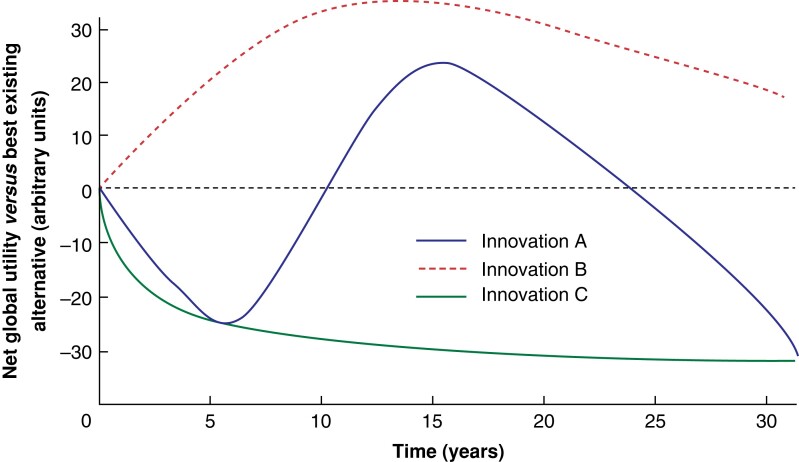
Net global utility compared with best existing alternative

For innovation B, only rudimentary alternatives exist. It has a low probability of causing harm. The surgical checklist is an example of innovation type B. Its utility is also unlikely to decline substantially as any alternative (a more comprehensive checklist) can have only very limited enhanced utility.

Innovation C is a highly complex intervention, which requires extreme skill or knowledge to use safely, such as laparoscopic abdominal aortic aneurysm repair^18^. It may have a very long learning curve. It may require a skill set that is diminishing in availability (open vascular surgery and high-level laparoscopic suturing skills) and it has more than negligible risks of catastrophic outcomes.

The process of innovation is always valuable and can lead to great advances, both intended and unanticipated. However, the outcomes must always be examined carefully in terms of metric, which considers more than could ever be tested in an RCT. Although an imperfect measure, net global utility offers an excellent method for assessing innovation.

## Data Availability

No new data were created or analysed in this study.
